# Biodegradability standards for carrier bags and plastic films in aquatic environments: a critical review

**DOI:** 10.1098/rsos.171792

**Published:** 2018-05-23

**Authors:** Jesse P. Harrison, Carl Boardman, Kenneth O'Callaghan, Anne-Marie Delort, Jim Song

**Affiliations:** 1 UK Centre for Astrobiology, School of Physics and Astronomy, University of Edinburgh, Edinburgh EH3 9FD, UK; 2 Division of Microbial Ecology, Department of Microbiology and Ecosystem Science, Research Network ‘Chemistry Meets Microbiology’, University of Vienna, 1090 Vienna, Austria; 3 School of Engineering and Innovation, The Open University, Milton Keynes MK7 6AA, UK; 4 Department for Environment, Food and Rural Affairs, London SW1P 3JR, UK; 5 Université Clermont Auvergne, Institut de Chimie de Clermont-Ferrand, CNRS, BP 10448, 63000 Clermont-Ferrand, France; 6 Wolfson Centre for Materials Processing, Brunel University, Uxbridge, UB8 3PH, UK

**Keywords:** biodegradability, plastics, carrier bags, standards

## Abstract

Plastic litter is encountered in aquatic ecosystems across the globe, including polar environments and the deep sea. To mitigate the adverse societal and ecological impacts of this waste, there has been debate on whether ‘biodegradable' materials should be granted exemptions from plastic bag bans and levies. However, great care must be exercised when attempting to define this term, due to the broad and complex range of physical and chemical conditions encountered within natural ecosystems. Here, we review existing international industry standards and regional test methods for evaluating the biodegradability of plastics within aquatic environments (wastewater, unmanaged freshwater and marine habitats). We argue that current standards and test methods are insufficient in their ability to realistically predict the biodegradability of carrier bags in these environments, due to several shortcomings in experimental procedures and a paucity of information in the scientific literature. Moreover, existing biodegradability standards and test methods for aquatic environments do not involve toxicity testing or account for the potentially adverse ecological impacts of carrier bags, plastic additives, polymer degradation products or small (microscopic) plastic particles that can arise via fragmentation. Successfully addressing these knowledge gaps is a key requirement for developing new biodegradability standard(s) for lightweight carrier bags.

## 1. Introduction

With a global estimate of over 300 million tonnes of plastic and a trillion plastic bags produced annually, the single-use carrier bag is a curiously iconic ambassador for polymer products [1,2]. Further to showing high service-life performance in terms of tensile strength and costs per unit weight, carrier bags are often considered an unwanted symbol of plastic pollution. This pollution is so ubiquitous that its presence can be used to mark the likely beginning of a new geological epoch called the Anthropocene [3]. Most of this pollution reaches marine environments through pathways including inland waterways [4,5]. Recent research suggests there are five trillion plastic pieces floating in the world's oceans collectively weighing over 250 000 metric tonnes, with fragmentation of larger items being a primary source of microplastic particles (less than 5 mm) [6]. Plastic is often also identified as the most abundant physical pollutant on the seafloor. For example, a coastal survey by Ioakeimidis *et al*. [7] found bags to account for approximately a third of this type of litter in terms of item abundance.

Further to concerns related to the abundance of plastic litter, the negative environmental image of carrier bags stems from evidence for the deleterious consequences of plastics within open (unmanaged) ecosystems [8]. In addition to mortalities caused by entanglement and suffocation, ingested plastics can transfer organic pollutants into food webs [9–12]. Alongside increasing recognition of the negative environmental effects of plastic items, the last 40 years have involved a movement towards developing ‘biodegradable’ plastics (§2 and [13,14]). One advantage of bio-based polymers is that they can provide an alternative to fossil fuel-derived plastics. Given the right conditions, they can also relatively easily be broken down into water, biomass and gas. Their application as an alternative to traditional polymers, however, is not without challenge [15]. Bio-based films have higher costs associated with production and reduced mechanical properties compared to conventional plastics. It has also been suggested that the widespread use of biodegradable plastic bags would at best have no effect on littering and at worst could exacerbate the problem by introducing partially decomposed materials into the environment, as well as reducing social responsibility, for example by reinforcing the perception that using these products minimizes or even negates the adverse environmental impacts of plastic waste [16–18].

Further to environmental issues, bio-based products need to be compatible with waste treatment and recovery processes. However, bio-based polymers are not universally compatible with these. For example, while poly(lactic acid) degrades sufficiently well to be incorporated into compost, under mesophilic anaerobic digestion conditions it remains largely intact [19,20]. Bio-based polymers are equally problematic for plastic film reprocessing operations where their detection and separation remain impossible [21,22]. The introduction of biodegradable films, therefore, requires an assessment of their biodegradability within both managed and natural environments using harmonized assessment criteria. In comparison with managed environments, however, few studies have investigated plastic biodegradability within open ecosystems (e.g. [23–25]). Moreover, few biodegradability standards have been developed with regard to plastic litter deposited in unmanaged natural environments.

Early work on the standardization of biodegradability test methods was carried out in the 1980s, for example by the Organisation for Economic Co-operation and Development (OECD) [26]. In 1996, the European Commission mandated the European Committee for Standardisation (CEN) to develop standards for packaging, including those for compostability (Mandate M/200). This mandate was linked to the 94/62/EEC Directive and led to the establishment of the BS EN 13432:2000 standard [27]. BS EN 13432:2000 [27] includes a requirement to measure microbial respiratory gas evolution to ascertain biodegradation and includes tests for disintegration of the test material, an assessment of heavy metal concentrations and a plant ecotoxicity test. Although BS EN 13432:2000 [27] was instrumental in establishing a system of standardization, product certification and compliance, no equivalents to this biodegradability standard currently exist for unmanaged natural environments. Indeed, evaluating the biodegradability of plastics within unmanaged ecosystems is highly challenging, not least because such work would ideally need to be accompanied by information on the long-term environmental impacts of the product in question.

There has been debate on whether tax exemptions for biodegradable materials should be granted from plastic bag bans and levies so that, alongside plastic bag reduction measures, opportunities to establish filmic materials with better environmental credentials might be supported. This was considered but rejected in Northern Ireland [28], and has been considered in England [29]. When considering the possibility of a biodegradability standard for plastic films in the open environment, reliance on robust evidence is critical, because such a ground-breaking standard may influence expectations of standardization for other products. For example, approximately half of current plastic products are disposable [21]. There is also considerable interest in many other degradable products likely to find their way into the environment. Efforts have been made, for example, to develop standards on the biodegradability of biolubricants [30]. Other examples of potentially biodegradable products include bottles, cutlery, hygiene products, agricultural equipment and fishing gear [31,32].

From both a standardization and nature conservation standpoint, the diversity of potentially biodegradable products underscores the need to tread carefully. An incentive to increase the distribution of one type of product deemed to be environmentally safe is likely to be followed by calls to approve other materials and products. It would be risky to give the impression through a formal standard that plastic biodegradation in the open environment is manageable if our underpinning knowledge still has significant gaps. There are several questions that should be considered in this context, including:

— Are there standards that could establish biodegradability product performance and safe environmental credentials for plastic films and bags that might be unintentionally littered?
— What are the strengths and weaknesses of current biodegradability standards and specifications for plastic films?
— Which results and conclusions can (or should not) be extrapolated from laboratory tests designed to simulate conditions in the open environment?
— What would need to happen to enable the development of a biodegradability standard specification, and a compliance and certification system, for a new generation of plastic bags claiming to be biodegradable in all open environments?
— Are all open environments equally well supported by biodegradability standards and scientific knowledge, or are some better served than others?

Here, we review what is known about the biodegradability of plastics to try to answer these questions, focusing on aquatic environments that serve as transportation pathways and sinks for the accumulation of plastic waste [4,5]. We discuss the current situation concerning biodegradability standards and highlight knowledge gaps that would have a bearing on the development of a plastic biodegradability specification for the open environment. Existing standards and specifications developed by several authorities (ASTM International; British Standards Institution, BSI; International Organization for Standardization, ISO; and Association Française de Normalisation, AFNOR) were reviewed. The scientific literature on the biodegradability of plastics was also reviewed, particularly where relevant to biodegradation in open environments. Several other channels of enquiry, through industry and standardization authorities, allowed a fuller understanding of the situation for plastic materials and their biodegradability performance.

## 2. Definitions of biodegradability

Plastic biodegradability science and standards cannot be considered without an understanding of the definitions that shape the development of biodegradability standards and specifications. One key weakness of the term ‘biodegradable’ is that it does not contain any information on the location, timescale and extent of the decomposition process. Indeed, biodegradability is often defined in relation to the purpose or the conditions of interest, with separate standards and test methods having been developed for aerobic and anaerobic wastewater, freshwater environments and marine habitats (§3.1.1 and §3.1.2). Several OECD classifications for biodegradability exist, depending on biodegradation test performance and whether the term is taken to mean complete mineralization by microorganisms or an alteration in the chemical structure of a material due to biological activity (summarized in [33]). There is also widespread public uncertainty concerning the exact meaning of this term. It is frequently conflated with ‘compostable' and ‘bio-based', with product labelling often a cause of additional confusion (see [14] for a detailed discussion). When considering managed biodegradation processes, such as industrial composting, a sufficient definition of biodegradability can be reached with relative ease because the process conditions can be agreed upon and standardized. However, great care must be taken when attempting to define biodegradability within open environments, where a broad and complex range of physical and chemical conditions is typically encountered [14].

For purposes of this review, we define a biodegradable compound as one that is completely used as a source of carbon for microbial growth (based on the OECD definition of ‘ultimate biodegradability'). Under aerobic conditions, the degradation process results in the production of carbon dioxide, water, mineral salts and new biomass. Under anaerobic conditions, methane and/or low-molecular-mass acids can also be produced.

## 3. Biodegradability in aquatic environments

### 3.1. Science and standards

#### 3.1.1. Freshwater and wastewater environments

Freshwater habitats include environments such as rivers, streams, lakes and wetlands. To our knowledge, no active international or regional-level biodegradability standards have been published with reference to plastic within these environments, including the water column and sediments. Similarly, no biodegradability specifications with pass or fail criteria have been established for the disposal of plastic items within open freshwater ecosystems, although a single conformity mark (Vinçotte OK Biodegradable WATER [34]) has been developed. Products complying with this conformity mark are described as biodegradable ‘in a natural freshwater environment', with accepted products required to exhibit a biodegradation rate of 90% at temperatures of 20–25°C and within 56 days of incubation. The biodegradability standards used as part of this certification are based on tests employing activated sludge, compost or soil as the inoculum (as opposed to field-collected freshwater or microbial strains isolated from freshwater environments). To our knowledge, no new standards are currently under development for assessing the biodegradability of plastics within unmanaged inland waterbodies.

In contrast with unmanaged freshwaters, several biodegradability standards and test methods have been developed for plastics within aerobic and anaerobic wastewater. Two currently active international standards exist for assessing the biodegradability of plastics within aerobic wastewater and sewage sludge: BS EN ISO 14851:2004 [35] and BS EN ISO 14852:2004 [36] (table 1). These standards are used as part of the Vinçotte OK Biodegradable WATER conformity mark [34] and the specification BS EN 14987:2006 [39], both of which share the same requirements for a material to be defined as biodegradable. Further to these, the withdrawn test methods ASTM D5209-92 [40], D5271-02 [41] and D6340-98(2007) [42] were developed to assess the biodegradability of plastics in the presence of aerobic wastewater and sewage sludge. ASTM D5209-92 [40] was procedurally similar to EN ISO 14852:2004 [36] and involved ‘obtaining activated sewage sludge from a municipal waste water treatment plant and preparing inoculum, exposing plastic material to the aerated inoculum, [and] measuring carbon dioxide evolved as a function of time, soluble organic carbon (SOC) content and residual polymer weight'. ASTM D5271-02 [41] was equivalent to BS EN ISO 14851:2004 [35], while ASTM D6340-98(2007) [42] involved measurements of ^14^CO_2_ evolution from radioisotope-labelled plastics with assays conducted at a temperature of 58 ± 5°C. To our knowledge, no new biodegradability standards or test methods are currently under development for assessing plastic biodegradation within aerobic wastewater and/or sewage sludge.

**Table 1.** Currently active biodegradability standards for plastic materials within wastewater and sewage sludge.

standard	inoculum	medium	temperature (°C)	measurement type	test duration	no. of experimental replicates	validity criteria
BS EN ISO 14851:2004 [35]	sludge, compost and/or soil	synthetic; aerobic	20–25 (± 1)	BOD^a^; static test conditions	max. six months	min. 2	greater than 60% degradation of reference material; BOD of negative control must not exceed a specified upper limit
BS EN ISO 14852:2004 [36]	sludge, compost and/or soil	synthetic; aerobic	20–25 (± 1)	CO_2_ evolution; static test conditions	max. six months	min. 2	greater than 60% degradation for reference material; CO_2_ evolved from negative control must not exceed a specified upper limit
BS ISO 13975:2012 [37]	sludge, livestock faeces or other organic waste	direct exposure to inoculum; anaerobic	35 ± 3 or 55 ± 5	CO_2_ and CH_4_ evolution, DIC^b^; static test conditions	max. three months	2	greater than 70% degradation of reference material after 15 days; extent of degradation (%) must differ by <20% between replicates
BS EN ISO 14853:2016 [38]	digested or laboratory-prepared sludge	synthetic; anaerobic	35 ± 2	CO_2_ and CH_4_ evolution, DIC^b^; static test conditions	max. three months	min. 3	greater than 70% degradation of reference material; pH of medium must remain between 6 and 8

^a^Biological oxygen demand.

^b^Dissolved inorganic carbon.

In addition to standards and test methods developed for aerobic wastewater, two international biodegradability standards exist for plastics within anaerobic wastewater and sewage sludge: BS ISO 13975:2012 [37] and BS EN ISO 14853:2016 [38] (table 1). A single withdrawn regional test method, ASTM D5210-92(2007), has also been published [43]. This test method was similar to BS EN ISO 14853:2016 [38], with the exception of involving measurements of SOC concentrations and polymer mass.

Both active and withdrawn standards and test methods developed for evaluating the biodegradability of plastics within freshwaters typically aim to assess ultimate biodegradability, involving measurements of CO_2_ and CH_4_ evolution. The tests are conducted under controlled laboratory conditions, frequently involving exposure of the test material to an artificially inoculated medium at a specific temperature range (minimal and maximal test temperatures of 19°C and 63°C, respectively). As part of a single standard (BS ISO 13975:2012) [37], the polymer is directly exposed to the inoculum (i.e. no synthetic growth medium is used). All procedures described for freshwater environments are completed using static exposure conditions, as opposed to flow-through or *in situ* conditions. Maximal test durations of six months are used, although no maximal duration is given in ASTM D5210-92(2007) [43].

The inocula specified in the test procedures for freshwater environments originate from several sources, ranging from wastewater and field-collected or laboratory-prepared sludge to soil, compost, livestock faeces or non-specific ‘organic waste' (table 1). The inoculum may involve a single or several environmental sources, and may be modified (e.g. washed or adjusted to a desired number of colony-forming units, CFU) and/or exposed to the test material prior to use. While the majority of standards and test methods for these environments do not recommend a specific cell density to be used, CFU counts of 10^3^–10^6^ ml^−1^ have been recommended by two standards: BS EN ISO 14851:2004 [35] and 14852:2004 [36].

For a single test method (ASTM D6340-98(2007)) [42], no guidelines are given concerning the required number of experimental replicates. Three of the standards developed for wastewater [35–37] require a minimum of two replicates with reference to the test material. The remaining test procedures involve at least three replicates. Most of the protocols recommend using powdered plastic, but other sample types (such as films, pieces and fragments) are also accepted. Further, although using additive-free plastics is recommended by certain protocols, the standards and test methods are typically stated to be suitable for a broad range of test materials (including copolymers and polymer mixtures, additive-containing plastics and water-soluble polymers).

#### 3.1.2. Marine environments

Marine environments cover two-thirds of the Earth's surface area and span a great diversity of habitats, including open-ocean and coastal ecosystems (such as estuarine and tidal sites), as well as deep-sea environments. Two active international standards have been published with reference to the aerobic biodegradation of plastics at the interface between seawater and sandy marine sediment (ISO 18830:2016 [44] and ISO 19679:2016 [45]; table 2). These standards involve laboratory-based biodegradation tests using measurements of oxygen demand or CO_2_ evolution. Additionally, two active regional test methods have been developed for assessing the biodegradability of plastic materials in aerobic seawater: ASTM D6691-09 [46] and D7473-12 [47] (table 2). ASTM D6691-09 [46] employs measurements of CO_2_ evolution under controlled conditions, and ASTM D7473-12 [47] is based on visual evidence for biodegradation and loss of polymer mass (%) following exposure to seawater in a flow-through system (table 2). The latter test method is considered insufficient for establishing biodegradability on its own, and is only completed for materials achieving at least 30% biodegradability in ASTM D6691-09 [46]. A test method has also been published for measuring the aerobic biodegradability of plastics under conditions simulating burial in sandy tidal sediments, using measurements of CO_2_ evolution (ASTM D7991-15 [48]; table 2). In this test method, plastic is exposed to field-collected sediment and seawater that have been stored in the laboratory for up to five weeks prior to use.

**Table 2.** Currently active biodegradability standards and test methods for plastic materials within marine environments.

standard or test method	inoculum	medium	temperature (°C)	measurement type	test duration	no. of experimental replicates	validity criteria
ISO 18830:2016 [44]	sediment or sediment and seawater	synthetic or natural seawater	15–28 (± 2)	BOD^a^; static test conditions	max. 24 months	3	greater than 60% degradation of reference material; BOD of negative control must not exceed a specified upper limit
ISO 19679:2016 [45]	sediment or sediment and seawater	synthetic or natural seawater	synthetic or natural seawater	CO_2_ evolution; static test conditions	max. 24 months	3	greater than 60% degradation of reference material; CO_2_ evolved from negative control must not exceed a specified upper limit
ASTM D6691-09 [46]	preselected strains or seawater	synthetic; aerobic	30 (± 1)	CO_2_ evolution; static test conditions	max. three months	NS^b^	greater than or equal to 70% degradation of reference material
ASTM D7473-12 [47]	seawater or a combination of seawater and sediment	direct exposure to inoculum; aerobic^c^	varies depending on *in situ* conditions	visual evidence for degradation; loss of dry mass	max. six months	3	NS^b^
ASTM D7991-15 [48]	sediment and seawater	direct exposure to inoculum; aerobic	15–28 (± 2)	CO_2_ evolution; static test conditions	max. 24 months	3	greater than or equal to 60% degradation of reference material

^a^Biological oxygen demand.

^b^Not specified.

^c^Although ASTM D7473-12 states that ‘anaerobic processes (e.g. sulfate reduction) can play a role in the biodegradation', the test material is placed on the sediment surface (which is in direct contact with oxygenated seawater).

The withdrawn test method ASTM D6692-01 [49] was developed to assess the aerobic biodegradability of plastics in seawater by measuring the evolution of ^14^CO_2_ from radioisotope-labelled polymers. Furthermore, the withdrawn specification ASTM D7081-05 [50] was designed for plastics within several marine environments and was stated to cover aerobic seawater as well as anaerobic sediments. Requirements within the specification included criteria for the degree of plastic disintegration during degradation, rates of biodegradation and ecotoxicological testing (toxicity tests employing microorganisms, fish, *Daphnia* sp. or algae). A conformity mark has also been developed for products described as biodegradable in seawater (Vinçotte OK Biodegradable MARINE [51]). The biodegradability component of this certificate is based on ASTM D7081-05 [50], with accepted products required to exhibit a biodegradation rate of 90% following six months of exposure. Requirements for test material disintegration and toxicity testing, based on ASTM D7081-05 [50], are also included. An incubation period of three months is required prior to the toxicity tests, with a further requirement for ‘less than 90% of the tested organisms' being affected.

In general, existing test methods for marine environments are similar to those published for wastewater and sewage in that they are primarily aimed at assessing ultimate biodegradability (§3.1.1). The test inocula may consist of pre-selected strains or taxa naturally present in the environment, with direct exposures to the inocula being included either as an option or a requirement in current test methods (table 2). Similarly to procedures published for freshwater habitats, the inocula used for marine biodegradation tests may originate from a single or several environmental matrices, with a limited selection of standards recommending CFU counts of 10^3^–10^6^ ml^−1^ for use. Minimal and maximal test temperatures of 13 and 30°C are employed, respectively, and most of the test methods employ maximal exposure durations of six months (although ASTM D7991-15 [48], BS ISO 18830:2016 [44] and BS ISO 19679:2016 [45] involve a maximal duration of 2 years; table 2). Moreover, test methods developed for marine environments are broadly comparable with those published for wastewater and sewage in terms of their requirements for experimental replication and validity, as well as polymer types and morphology (table 2 and §3.1.1). ASTM D7991-15 [48], however, recommends evaluating the test materials as films rather than as powders.

Although biodegradability standards and test methods have been published for plastics deposited at the seawater–sediment interface and within aerobic sand (table 2), no current standards or specifications are specifically aimed at assessing the biodegradability of plastics within anaerobic marine habitats, saltmarshes, brackish waters, or cold and nutrient-poor deep-sea environments. Although low-oxygen environments were nominally included in ASTM D7081-05 [50], the procedures listed in this document were based on the addition of inocula into aerobic aqueous media, as opposed to using strictly anaerobic exposure conditions.

### 3.2. Standardization issues

While a number of standards and test methods for measuring the biodegradability of polymers within aquatic environments have been developed, several issues can limit their reliability when attempting to predict *in situ* rates of biodegradation. These issues can be summarized as uncertainties originating from (i) approaches to inoculum preparation and test conditions, (ii) a lack of specific guidelines for employing different test materials, (iii) insufficient statistical replication, (iv) a lack of suitable procedures for unmanaged aquatic environments, and (v) deficiencies in toxicity testing and our understanding of the wider impacts of plastic litter on aquatic ecosystems.

#### 3.2.1. Preparation of inocula and test conditions

Sources of inocula recommended for aquatic biodegradability tests are diverse and often non-specific. The type of inoculum and the way in which it is prepared can significantly affect rates of biodegradation [52–56]. For example, filtration can alter the taxonomic diversity of an inoculum, which can result in reduced test reliability and difficulties in extrapolating from laboratory-based data to patterns of biodegradability within natural environments [55]. Inoculum storage times specified by current test procedures are often also unclear, despite the potential for prolonged sample storage to result in experimental bias [57–59]. Furthermore, little information is frequently provided on recommended cell densities, despite evidence that the concentration of the inoculum in relation to the test material can affect the results of biodegradability tests [54,56].

Using preselected or preconditioned strains, as well as synthetic media and static exposure conditions constitutes further sources of uncertainty when employing existing test procedures to assess the biodegradability of plastics within open aquatic environments. These uncertainties are well recognized in the scientific literature [60,61], with the need for updated test procedures having been highlighted by several authors [55,56,62,63]. Only a limited number of existing biodegradability test procedures for aquatic habitats involve direct exposure of the test material to the inoculum, and/or flow-through conditions.

The temperature ranges employed by published biodegradability tests and guidelines are limited, and typically do not account for seasonal fluctuations in environmental conditions which can influence the composition and activities of plastic-associated microorganisms [64–68] (but see ASTM D7473-12 [47]; table 2). As such, temperatures employed by existing procedures are likely to be unsuitable for assessing the biodegradability of plastics within (temperate) unmanaged waters, where temperatures can be considerably lower than 13°C (the lower temperature limit for current test procedures; ISO 18830:2016 [44] and ASTM D7991-15 [48] in table 2). Carrier bags are likely to exhibit lower rates of biodegradation within unmanaged waters in comparison with wastewater and environments such as compost (owing to differences in both the environmental conditions and taxa present), with certain polymer types having been predicted to require decades or centuries to become fully biodegraded within marine ecosystems [17,23,61,69,70]. The maximal durations of current biodegradability tests, therefore, are likely to be insufficient for assessing polymer biodegradation within several habitats (e.g. lake bottoms and deep-sea habitats characterized by low temperatures, as well as low availabilities of oxygen, nutrients and light) [23,61,71,72].

#### 3.2.2. Lack of specific guidelines for different test materials

Most test procedures described for aquatic environments are stated to be suitable for composite materials, including polymer blends and plastics that contain additives. Even so, while rates of biodegradation can considerably vary between polymer types [73], none of the published procedures provide clear instructions for accounting for this or the impacts of additives on biodegradation rates. The latter issue, however, is recognized by the standards BS EN ISO 14851:2004 [35] and 14852:2004 [36]. Many of the test procedures also rely on single measurement methods, which can lead to unreliable results when assessing the biodegradability of composite materials [74]. Although measurements employing ^14^C-labelled polymers can avert these issues [75,76], they are likely to be unsuitable for wide-scale use due to the high cost of radioisotope-labelled materials, and potential problems concerned with licensing and/or waste disposal [75].

In addition to these drawbacks, current standards and test methods for aquatic environments do not provide clear guidelines for assessing the biodegradability of materials of different shapes and sizes, despite this having been recognized as important (BS EN ISO 14851:2004 [35] and 14852:2004 [36]). Materials with a comparatively high surface area to volume ratio (e.g. powders) have been shown to exhibit higher rates of biodegradation than other materials (including films), which can result in an overestimation of biodegradation rates [77]. As a further source of uncertainty, the relationship between the shape and size of a material and its biodegradability can vary between polymer types and test conditions [77]. Crucially, the surface properties of a material (such as its roughness) can also influence the results of biodegradability tests [66,67].

#### 3.2.3. Insufficient statistical replication

Many of the biodegradability standards developed for aquatic environments are either characterized by a lack of information on the level of statistical replication required, or involve measurements performed in duplicate (tables 1 and 2). A minimum of triplicate measurements is required to ensure statistical validity and reproducibility as part of a given biodegradability standard.

#### 3.2.4. Lack of suitable procedures for unmanaged aquatic environments

There is an absence of standards and test methods for evaluating the biodegradability of plastic materials within unmanaged freshwater ecosystems (including lakes, streams and rivers), and most marine environments [63]. The test temperatures used by existing procedures, as discussed above, are frequently higher than those encountered within (temperate) aquatic environments, despite the ability of temperature to strongly influence the taxonomic composition and metabolic activities of microbial communities [61,66,67,78]. Furthermore, the maximal test durations of published test procedures are likely to be too low for assessing the breakdown of certain polymer types within several unmanaged aquatic ecosystems, due to rates of biodegradation being comparatively low within these environments (although maximal exposures of 2 years are included as part of three marine standards and test methods; table 2). Indeed, limitations of laboratory-based test procedures for estimating the biodegradability of plastics within unmanaged ecosystems are explicitly acknowledged in ASTM D6691-09 [46]: ‘it shall be recognized that predicting long-term environmental fate and effects from the results of short-term exposure to a simulated marine environment is difficult. Thus, caution shall be exercised when extrapolating the results obtained from this or any other controlled environment test to disposal in the natural environment*'*.

As an example of plastic biodegradation rates estimated for open aquatic environments, a lifespan of approximately 10 years has been calculated for polyhydroxybutyrate–polyhydroxyvalerate (PHBV) bottles on the surface of lake sediments at a depth of 85 m and at 6°C [79]. In this same study, biodegradation rates on the sediment surface were also found to be approximately twice lower than those at a water depth of 20 m (measured as mass loss). Moreover, an analysis of plastic fragments recovered from the sediments of Lake Ontario (Canada) suggests that these materials have been accumulating in the lake bed for approximately 40 years [80]. Given this evidence for the multi-decadal preservation of plastic in freshwater sediments, it is likely that several polymer types, including bioplastics, will require unprecedented amounts of time to biodegrade within these and several other benthic habitats. Indeed, rates of polymer biodegradation within marine ecosystems have often been found to be lower than those estimated for both freshwater and terrestrial environments [23,61,81].

#### 3.2.5. Toxicity testing and wider environmental impacts

With exception of the Vinçotte OK Biodegradable MARINE conformity mark [51], toxicity tests for plastics, plastic additives or polymer degradation products are not included as part of active biodegradability standards or test methods developed for aquatic ecosystems [14]. Where toxicity assays are required, these are based on single test methodologies. Additionally, one of the toxicity tests recommended by the Vinçotte OK Biodegradable MARINE label [51] (as well as the withdrawn specification ASTM D7081-05 [50]) is based on *Daphnia* sp., a genus of freshwater crustaceans, despite this conformity mark having been developed for marine environments. The impacts of plastic litter on multispecies communities and biogeochemical processes, such as elemental cycling, are not addressed by existing test procedures [64,82]. Moreover, the toxicity assays required by the OK Biodegradable MARINE label [51] do not explicitly account for the ability of micro- or nanoscopic plastic particles to adversely affect aquatic organisms. For example, the presence of both conventional (polyethylene and poly(vinyl chloride)) and bio-based (poly(lactic acid)) polymer microbeads has been found to affect the feeding behaviour of the lugworm *Arenicola marina* [82,83]. Exposure to polystyrene nanoplastics has been shown to reduce population growth in the algal species *Scenedesmus obliquus*, and to affect the body size and reproductive behaviour of *Daphnia magna* [84]. Further, exposure to polystyrene microplastics has been found to influence the feeding behaviour and reproductive output of the copepod *Calanus helgolandicus* [85].

### 3.3. Knowledge gaps

We have identified several knowledge gaps with reference to the biodegradability of plastics within aquatic environments, which are summarized in the following. Where possible, recommendations are also provided for developing novel biodegradability standards for lightweight plastic carrier bags.

Knowledge gap 1: Relation of laboratory-based data to patterns of biodegradability within open aquatic environments

Most existing standards and test methods for assessing the biodegradability of plastic materials in aquatic environments are based on laboratory experiments that use respirometric measurements under idealized conditions. Measurements of CO_2_ and CH_4_ evolution can provide reliable estimates of biodegradation in these and other environments, as long as gas evolution from organic matter and other potential sources of uncertainty are accounted for [86–88]. However, current procedures are insufficient in simulating conditions encountered within natural environments, as well as the ability of these conditions to vary seasonally and geographically.

Many of the protocols published for marine environments do account for relatively complex exposure scenarios, such as those involving flow-through conditions, *in situ* temperatures and/or exposure to field-collected water or sediment (table 2). Certain aspects of these test procedures could provide a starting point for new methodological approaches that mimic key physical and chemical conditions encountered within aquatic environments (e.g. mesocosm experiments involving low temperatures and/or nutrient concentrations). The microbial taxa and abundances used in such biodegradability tests should be similar to those present in the environment of interest, and direct exposures to the inoculum are recommended in addition to strictly controlled experiments involving synthetic media. The inocula should also be used as soon as possible following their collection, and it is recommended that any material be tested as intended for public use (e.g. as intact carrier bags; where this is impossible, an attempt should be made to predict the lifetime of the intact product). Improved guidelines are additionally required for assessing the biodegradability of polymer composites and additive-containing materials, particularly as respirometric data can be poorly correlated with declines in the molecular weight of plastic blends [14,74].

Given extremely slow rates of polymer biodegradation reported by several field studies (e.g. [69,79]), it is likely that currently specified test durations will be insufficient for evaluating the biodegradability of polymers within many unmanaged aquatic habitats. Biodegradation rates are strongly dependent on the surrounding environmental conditions, and there is a need for wider research into the long-term fate of plastic materials within environments such as deep and nutrient-poor marine waters, and sediments subjected to cold temperatures [89].

Knowledge gap 2: Absence of biodegradability standards and test methods for unmanaged aquatic environments

Despite records of plastic debris in rivers, lakes, coastal waters and sediments, polar waters and the deep sea [6,18,80,90,91–95], there are no standards or test methods for evaluating the biodegradability of polymers in unmanaged aquatic environments other than open seawater and sandy sediments (table 2). As sediments represent an important sink for the accumulation of plastic litter, including materials that lose their buoyancy over time [18,90,91], there is a particular need to develop further standards for benthic aquatic environments. As rates of biodegradation on the sediment surface may not be representative of those within the sediment [72], this should also be considered by any new standard(s).

Knowledge gap 3: Lack of wider laboratory- and field-based research into polymer biodegradation within several aquatic environments

The successful design and implementation of new biodegradability standards for marine and freshwater habitats are severely hindered by a lack of relevant primary research [63]. For example, there is a need for further investigations of plastic biodegradation under exposure scenarios involving low temperatures and nutrient availability, as well as anaerobic conditions. To fully characterize the biodegradability of polymers within open environments, a combination of laboratory- and field-based research will be required. One potentially useful way to approach this topic could involve calculating ‘environmentally degradable parameter' (^Ed^*K*) values for several types of plastic exposed to a broad range of environmental conditions [73].

Knowledge gap 4: Toxicity tests and research into the wider impacts of plastic materials on aquatic ecosystems

Research into the toxicological and wider environmental impacts of a given test material will be required to identify acceptable time frames for the biodegradation of plastic waste in open (including aquatic) habitats. While some of the toxicity tests specified by the Vinçotte OK Biodegradable MARINE certificate [51] could be useful for developing a biodegradability standard(s) for lightweight carrier bags, they focus on a limited range of species and are conducted on the population (rather than community) level.

Further toxicological research is required particularly with reference to species that have been found to ingest plastic particles, including deposit- and filter-feeding invertebrates at the base of aquatic food webs [83–85]. Assessing the impacts of discarded plastic items on multiple species and community-level processes (such as microbially mediated elemental cycling) is also required to fully understand the environmental implications of aquatic plastic pollution. Indeed, despite several recent studies of biofilm formation on plastic debris in freshwater and marine environments [68,96–99], little is still known about how this debris affects ecosystem functioning (but see [82]).

## 4. Alternative test methods for measuring biodegradability

Test procedures for measuring polymer biodegradation are commonly based on measurements of biological oxygen demand or the evolution of CO_2_ and/or CH_4_ (tables 1 and 2). An alternative method based on ATP measurements has been proposed to assay the biodegradability of ‘oxo-degradable' polymer films [100]. Research related to the ANR OXOMAR Project DS0103 (*Abiotic and biotic degradation and toxicity of oxo-biodegradable plastics in marine waters*) is currently aiming to adapt this test to the marine environment. Simpler test methods using changes in physical properties of polymers as indicators for biodegradation are also widely used in the research literature and such methods are/were recommended by a selection of ASTM test methods:

— *Changes in visual appearance* are assessed as part of ASTM G21-15 [101] and the withdrawn test method G22-76(1996) [102] to evaluate resistance of plastic materials to fungi and bacteria, and in D7473-12 [47] for visual evidence of biodegradation in seawater, or seawater and sediment (*preferably muddy*).
— *Changes in mass* are monitored in the withdrawn ASTM test methods D5247-92 [103] and D6003-96 [104], as well as ASTM D7473-12 [47].
— *Changes in mechanical properties* are additionally assessed as part of ASTM D5247-92 [103].

These changes take place during different stages of biodegradation and thus it is important to identify stages when such physical changes are best to measure, and what adequate information they can provide on the breakdown of different polymers. The biodegradation process is often divided into three phases. When measurements of CO_2_ or CH_4_ evolution are used, gas evolution is negligible during early stages of biodegradation (lag phase) and subsequently exhibits a substantial increase (biodegradation phase), followed by a plateau phase when biodegradation is close to completion. These phases roughly correspond to distinct steps involved in the degradation process, as described in Lucas *et al*. [76] and Eubeler *et al*. [89,105]. The *biodeterioration stage* is dominated by depolymerization of the material by either enzymatic hydrolysis (e.g. ester and amide bonds) or peroxidation of carbon chain polymers. The *biofragmentation stage* results in disintegration and fragmentation of the material without significant gas evolution. Finally, the *microbial assimilation stage* corresponds to digestion of the low-molecular-weight species produced during earlier stages, resulting in significant gas evolution and mineralization.

**Figure 1.**
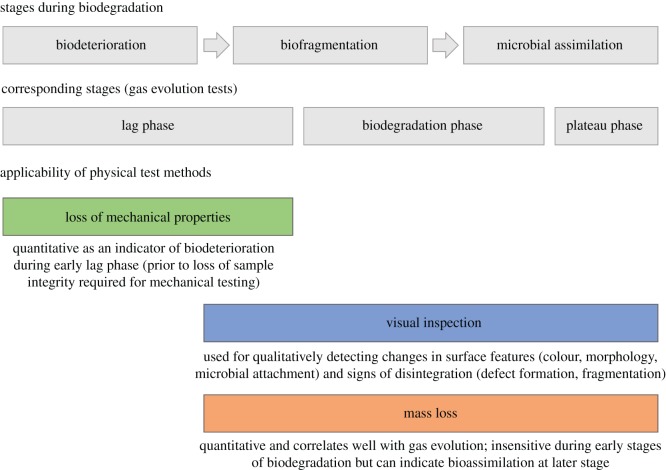
Physical test methods for the evaluation of plastic biodegradation, and their relation to different stages of polymer breakdown and corresponding stages observed using gas (CO_2_ and CH_4_) evolution tests.

Changes in the mechanical properties of a material often occur during the biodeterioration stage and the onset of fragmentation (figure 1). These are accompanied by changes in visual appearance and in mass loss as biodegradation proceeds, providing a first-order estimate of biodegradation (figure 1). Despite such physical tests being useful due to their low cost and simplicity, several issues limit their ability to provide conclusive information on the biodegradability of plastic materials. Visual inspection is qualitative and subject to considerable human error (e.g. due to variation in experience). Additionally, evidence for changes in surface morphology or the degree of microbial attachment are not indicative of biodegradation in their own right [105]. In contrast with visual inspection, measurements of mass loss are quantitative and generally correlate well with CO_2_ evolution [106]. However, as measurements of mass loss are performed at specific time intervals (rather than continuously) and are subject to inherent biases (detailed below), this correlation is comparatively poor during the early lag phase (figure 1). Materials exhibiting over 90% of mass loss are often assumed to be nearly completely biodegraded.

While measurements of mass loss are easy to perform in comparison with gas evolution methods, care must be taken with respect to several aspects. Many bioplastics, particularly those based on starch, are hygroscopic and the influence of changes in moisture on mass loss must be eliminated via drying the sample prior to measurements. As the moisture level is crucial to microbial activity, the drying step may interrupt biodegradation each time a sample is processed for analysis. This may be mitigated by a sacrificial sampling regime in which separate samples are analysed at each sampling interval. During degradation, fragmentation of materials may lead to the liberation of particles to the surrounding medium [14]. Absorption of diverse compounds from environmental matrices or the growth medium may also occur. While these can influence changes in mass, they can be partially counteracted by using containers (such as nylon mesh bags) [106] to separate the materials without preventing migration of moisture and microbial cells.

Like alterations in the visual appearance and the mass of a given material, changes in mechanical properties (e.g. tensile strength, elastic modulus and elongation at break) are simple to measure in comparison with gas evolution. These changes may be attributable to a reduction in molecular weight due to enzymatic hydrolysis or oxidation, and are therefore used as an indicator of the onset of biodegradation. However, even prior to significant biologically induced fragmentation, the formation of cracks and surface irregularities due to abiotic processes can severely compromise the reliability of mechanical test results. For example, certain (e.g. starch-based) bioplastics can become brittle with time due to a change in crystallinity [107]. Exposure to UV radiation and heat can also affect mechanical properties. This can make it difficult to correlate changes in mechanical properties with biodegradation.

To overcome limitations of the physical methods described above, they can be combined with other analytical techniques and approaches to confirm changes in the molecular structure of polymers during biodegradation. These include measurements of surface hydrolysis, chromatographic analyses and several spectrometric techniques (table 3). Optical and scanning electron microscopy (SEM) can also be used to assess the biodeterioration of the surface due to microbial activity or biofilm formation [115,117]. Although SEM and optical microscopy are not quantitative, fluorescence microscopy can be used to quantify microbial colonization of the polymer surface [119].

**Table 3.** Analytical techniques and approaches that can be used to supplement physical test methods for assessing the biodegradability of polymers.

technique or approach	application	references
surface hydrolysis measurements	monitoring release of hydrogen ions during aerobic biodegradation to provide information concerning sample degradation by selected enzymes	[108,109]
gas chromatography (GC) with flame ionization detection (GC-FID) or mass spectrometry (GC-MS)	analysis of the oligomeric fraction formed during polymer degradation; detection of low-molecular-weight metabolites or degradation intermediates	[110,111,112]
liquid chromatography–mass spectrometry (LC-MS)	analysis of complex oligomeric mixtures during biodegradation	[112]
gel-permeation chromatography (GPC)	measurements of molar mass and shifts in molecular weight	[105,113]
high-performance liquid chromatography (HPLC)	identification of individual homologues of low-molecular-weight polymers	[114]
nuclear magnetic resonance (NMR) spectroscopy	analysis of changes in polymer chain structure during degradation; monitoring biodegradation of soluble compounds released during incubation	[112,115,116]
Fourier-transform infrared (FT-IR) spectroscopy	analysis and quantification of specific functional groups (e.g. carboxylic and alcohol functional groups); carbonyl index measurement as a proxy of surface oxidation; monitoring of biofilm formation	[100,117,118]

Further information concerning analytical techniques to assess abiotic and biologically induced degradation processes is given in Lucas *et al.* [76] and Koutny *et al*. [117]. One limitation of many of the methods discussed above is that they can be expensive to carry out routinely. Measurements of surface oxidation based on the carbonyl index cannot be performed for all polymer types, as certain plastic materials are poorly predisposed to oxidation. Additionally, changes in the degree of surface oxidation may be due to abiotic (chemical, photochemical and thermal) and/or biotic (enzymatic) processes. When assessing the biodegradability of plastics within complex environments, it is likely that physical and mechanical tests (as well as measurements of the carbonyl index) will need to be complemented by other approaches, such as measurements of gas evolution.

## 5. Use of biodegradability standards in the research literature

Many of the experimental approaches described in biodegradability standards and test methods (tables 1 and 2) have been used in primary research. Some studies have been comparative, involving either a range of materials or a comparison of standardized methods [53,120,121]. However, to our knowledge, the only study to date that has attempted to extend standard test procedures into the open environment was performed by Tosin *et al.* [63], who developed new methods to evaluate the biodegradation of polymers within intertidal sediments and on permanently inundated nearshore sediments. In the former method, field-collected sandy sediment and seawater were stored within a sealed polypropylene box with air vents. Samples of Mater-Bi® film were buried into the sediment, followed by visual assessment of biodegradation. The latter method was a modification of the standard BS EN ISO 14851:2004 [35] (table 1), with the test vessels containing 15 g of wet sediment covered with 75 ml of artificial seawater. Samples of film were placed on top of the sediment and oxygen consumption was monitored by respirometry. Both tests were conducted at room temperature. Using these exposure conditions, Mater-Bi^®^ was shown to disintegrate within nine months (simulation of intertidal zone) and exhibit a biodegradation rate of 69% within approximately 240 days (inundated sediment) [63]. However, the test conditions employed in this work did not reflect many of the conditions that are often encountered in the marine environment (such as cold temperatures), and we are aware of no studies offering a fully robust protocol for applying existing standards to evaluate the biodegradability of plastics within unmanaged ecosystems.

Even when biodegradation within a given environment has been partly demonstrated, many investigators still regard long-term (complete) biodegradation as an unresolved issue [62,122]. Standards and specifications designed to measure biodegradability in environmental simulations often include a caveat on the need for additional corroborative studies to demonstrate an acceptable degree of biodegradability in the open environment. No standards, however, appear to give any guidance on how that corroborative work should be completed.

## 6. Conclusion

The conclusion of this review is that current international standards and regional test methods are insufficient in their ability to realistically predict the biodegradability of carrier bags within wastewater, inland waters (rivers, streams and lakes) and marine environments. This is due to several shortcomings in existing test procedures, the absence of relevant standards for the majority of unmanaged aquatic habitats, as well as a paucity of wider research into the biodegradation of plastic materials within these environments.

While most existing test procedures employ a reliable method for determining biodegradability (i.e. measurements of CO_2_ and CH_4_ evolution; tables 1 and 2), the data obtained by current standards, test methods and specifications can significantly underestimate the durations required for polymer biodegradation within natural ecosystems. This is partially due to biases associated with the preparation of experimental inocula and the test conditions themselves, including the use of preselected and/or pre-conditioned strains, artificially modified inocula, powdered test materials, nutrient-rich synthetic media and test temperatures that are frequently higher than those encountered within the environment. A key methodological issue associated with existing procedures is the lack of clear guidelines for the analysis of different polymer types, including composite materials and plastics that contain additives. There is also a paucity of guidelines for materials of varying shapes and sizes and, in certain cases, the test procedures lack a sufficient level of statistical replication. Additionally, no biodegradability standards or test methods for plastics have been published for unmanaged freshwater environments, or marine habitats other than open seawater, aerobic sandy sediment and the seawater–sediment interface.

Based on previously published research into plastic biodegradation within aquatic environments, the timescales required for polymer degradation are both highly variable and dependent on the surrounding conditions (including temperature and nutrient availability). For example, while the complete degradation of certain bioplastics is likely to require several months within wastewater treatment facilities, years or even decades may be required for PHBV bottles to undergo complete biodegradation following their deposition into deep lake environments [79]. Rates of polymer biodegradation within aquatic habitats have typically been estimated using indirect methods (such as mass loss), and there is a lack of research into this topic with reference to several environments (including deep seawater and cold marine sediments). Given the global-scale accumulation of plastic debris in rivers, lakes, coastal waters and sediments, polar waters and the deep sea, there is a requirement for further experimental data on polymer biodegradation within the majority of unmanaged aquatic ecosystems.

In addition to these issues, the toxicological and wider environmental impacts of aquatic plastic debris remain insufficiently understood, despite the requirement for toxicity testing by the Vinçotte OK Biodegradable MARINE conformity mark [51]. Indeed, no current standards or test methods account for the potential adverse impacts of plastics, plastic additives and/or polymer degradation products on deposit-feeding organisms at the base of aquatic food webs (such as marine worms [83]), or ecological processes such as microbially mediated elemental cycling. To facilitate the development of a new biodegradability standard(s) for plastic materials, there is a need for improved experimental methodologies that simulate key properties of aquatic ecosystems (including low temperatures and nutrient availability). For example, ISO 9887:1992 [123], ISO 14592-2:2002 [124] and ASTM D7473-12 [47] involve using a semi-continuous activated sludge method or flow-through system and could be adapted for further method development, including the design of biodegradability assays for aquatic environments not considered by current standards and test methods.

Finally, the interconnected nature of freshwater and marine ecosystems should be taken into consideration when developing novel approaches to assess the biodegradability of plastic materials. As environments such as wastewater treatment facilities and rivers act as important conduits for the transport of plastic litter to marine habitats [5], the anticipated retention time of the test material within the environment of interest will be a key topic to consider when determining a suitable test duration (§3.3). Products that pass a biodegradability test that has been exclusively developed for freshwater environments may still exhibit extremely slow rates of biodegradation if they become deposited within marine habitats, with direct repercussions for their environmental impacts and fate [16].

## Data accessibility

No new data have been generated for the purposes of this review article, and all data in this review are available from the sources referenced.
